# Visualization data on concentrating apple juice with a trinitarian crystallization suspension freeze concentrator

**DOI:** 10.1016/j.dib.2019.104155

**Published:** 2019-06-15

**Authors:** Frank G.F. Qin, Zhongxiang Ding, Jiaojiao Yuan, Runhua Jiang, Simin Huang, Huibin Yin, Youyuan Shao

**Affiliations:** a Guangdong Provincial Key-laboratory of Distributed Energy Systems, Dongguan University of Technology, Dongguan 523808, China; b School of Chemical Engineering and Light Industry, Guangdong University of Technology, Guangzhou, China

**Keywords:** Suspension crystallization, Freeze concentration, In-situ wash column, Apple juice, Ice slurry

## Abstract

This article contains visualization data on concentrating apple juice with a trinitarian suspension crystallization freeze concentrator, which integrates scraped-surface heat exchanger, suspension crystallizer and wash-column into one piece of equipment. The visualization data on ice accumulation, ice bed development and consolidation in the crystallizer/wash-column of the freeze concentrator are presented in a set of photographs in chronological order and videos attached as appendix materials. These data refer to the related research article entitled “Concentration of Apple Juice with an Intelligent Freeze Concentrator” Ding et al., 2019.

**Table:** Specifications table

Subject area	*Food Science and Technology*
More specific subject area	*Liquid food treatment and processing*
Type of data	*Photograph, video and real-time recorded data*
How data was acquired	*Photos and movies were taken during the experiment with the Freeze concentrator.*
Data format	*Raw and processed*
Experimental factors	*The apple juice was freeze concentrated with a prototype of intelligent freeze concentrator; The suspension crystallizer converted to a wash column at the end of freezing stage.*
Experimental features	*The apple juice of 10.5 °Bx was concentrated to 18–20 °Bx in a single pass of freeze concentration trial, meanwhile produced a porous packed ice bed with a porosity of approximately 50%, which was saturated with the concentrated mother liquor.*
Data source location	*Dongguan University of Technology, Dongguan, China, north latitude 23.16° and east longitude 113.23°*
Data accessibility	*Raw data is presented in this article*
Related research article	Z. Ding, F.G.F. Qin, J. Yuan, S. Huang, R. Jiang, and Y. Shao, Concentration of apple juice with an intelligent freeze concentrator. Journal of Food Engineering, 2019. 256: p. 61–72.

**Table:** 

**Value of the data** • The visualization data demonstrated how the ice slurry was primarily produced, as shown in Fig. 1(a) and (b), and video 1; how the ice slurry layer dynamically developed into a porous ice column (or ice bed), as shown from Fig. 1(c)–(e), and videos 2 and 3; how the ice crystallizer finally converted to an in-situ wash column, as shown in Fig. 1(f), and videos 4 and 5. • The visualization data of ice slurry, which is rotating in the crystallizer with a speed of slightly lower than the scraper of scraped-surface heat exchanger in approximately 100 rpm for Ostwald ripening, can be served as a benchmark for ripening the primary ice slurry produced by subcooled scraped surface heat exchanger, and will be useful for other researchers who is working on the field of freeze concentration and freeze separation by the suspension crystallization method. These can be seen in videos 1, 2 and 3. • The videos 1, 2 and 3 also show that the ice layer stays in the upper section of the crystallizer with a clear and sharp lower bottom, indicating the upward flow, which is driven by an external circulation, is reasonably good in pushing the ice up to form a porous packed ice bed, so an additional force to compress the ice bed, such as using a perforated piston to squeeze the ice bed is not necessary, this may give an impetus for the further development of freeze concentration system. • The behavior and the flow pattern of the ice slurry shown in video 1, 2 and 3, which is a two-phase fluid, in the crystallizer could be useful for those who is in the fields of freeze concentration, desalination, ice thermal storage, and high efficiency ice cooling with pumpable ice slurry. • The formation process of the porous packed ice bed and the separation of the concentrated mother liquor from the ice bed by washing may be useful for other innovative emerging technology.

## 1 Data

**Fig. 1:**
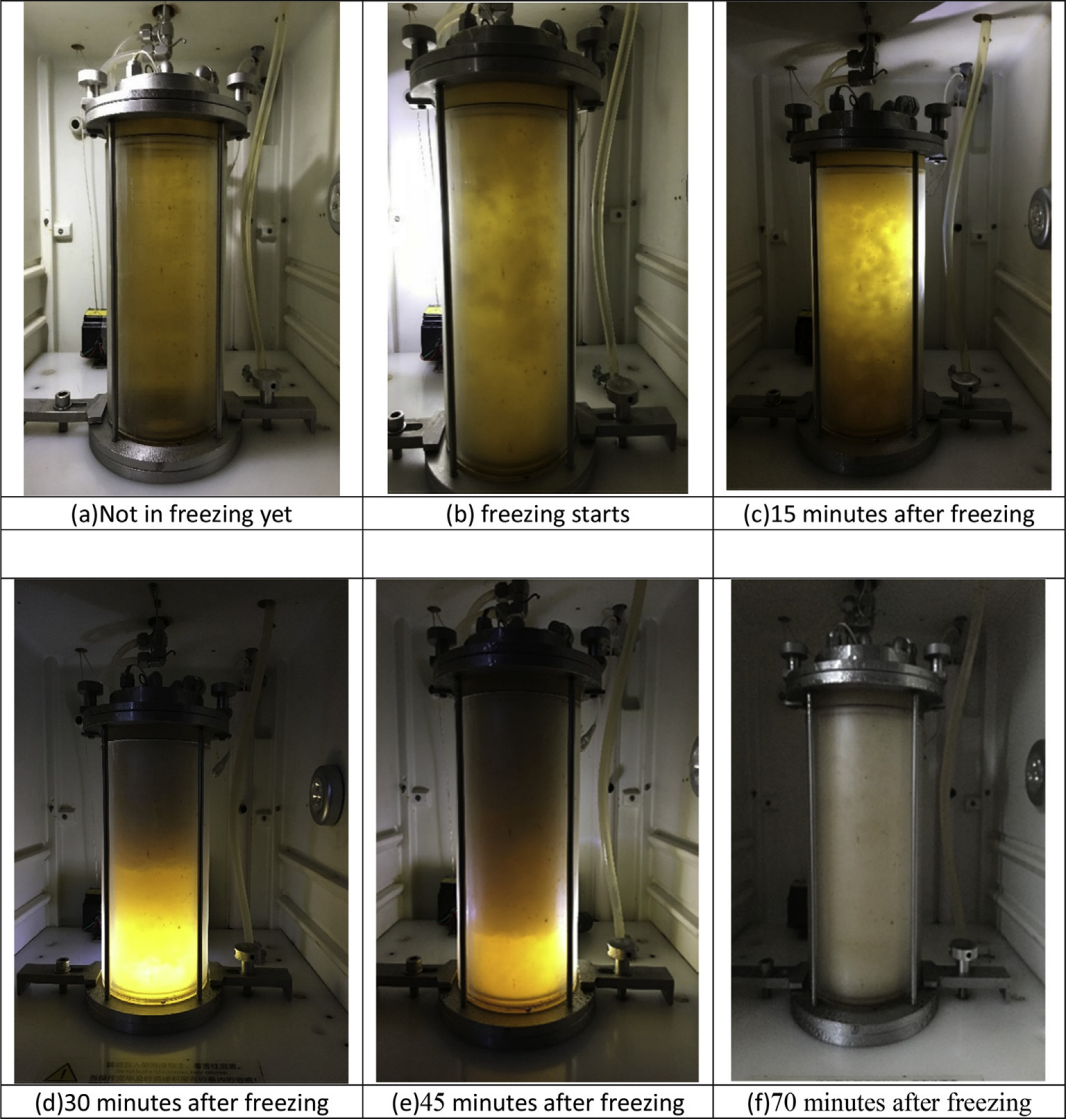
The appearance, accumulation of floating ice particles and the formation of a packed ice bed in the suspension crystallizer, which converts to a wash column at the end of freezing stage.

Fig. 1(a) shows the apple juice in cooling stage, during which there is no ice in the solution. Fig. 1(b) shows the appearance of the packed ice bed in the suspension crystallizer used for freeze concentration, Fig. 1(c)–(e) shows the development of packed ice bed. Fig. 1(f) shows that the crystallizer converts to an in-situ wash column for separating the ice and concentrate at the end of freezing.

The video clips of.

Appendix A video 1 Ice bed appears.mp4;

Appendix A video 2 Ice bed builds up 1.mp4;

Appendix A video 3 Ice bed builds up 2.mp4;

Appendix A video 4 Final packed ice bed 1.mp4;

Appendix A video 5 Final packed ice bed 2.mp4.

provide the visualization data of the formation of the packed ice bed in the freeze concentrator. They have been edited to give captions.

The following are the supplementary data related to this article:

2

3

4

5

6

Appendix B 2017080301.xls, this Excel file gives the raw data of one of the experimental trials of freeze concentration of apple juice, which was exported by the controller of the instrument and recorded by a computer in real time.

## 2 Experimental design, materials, and methods

The apple juice used in this study was Huiyuan^®^ brand (China Huiyuan Juice Group Ltd., Beijing, China) purchased in a local market.

The apple juice was cooled and frozen to produce ice slurry by a scraped-surface heat exchanger below a 2.5-L suspension crystallizer. As the content of ice accumulated in the apple juice, a tightly packed ice bed was formed at the end, and the crystallizer then converted to a wash column that was used for separate the ice with the concentrated apple juice [1]. The separation of ice and concentrate is performed by in-situ top downwards wash of the ice with 0 °C water to displace the concentrate. The freezing point depression (FPD) data of sixteen liquid were saved in the controller. It can therefore handle several commonly seen liquid food.
